# **Microbial ecology of the cryosphere (glacial and permafrost habitats): current knowledge**

**DOI:** 10.1007/s00253-019-09631-3

**Published:** 2019-02-05

**Authors:** Rosa Margesin, Tony Collins

**Affiliations:** 1 Institute of Microbiology, University of Innsbruck, 6020 Innsbruck, Austria; 2 Centre of Molecular and Environmental Biology (CBMA), Department of Biology, University of Minho, 4710-057 Braga, Portugal

**Keywords:** Psychrophiles, Permafrost, Cryoconite, Glacial, Climate change, Biodiversity

## Abstract

Microorganisms in cold ecosystems play a key ecological role in their natural habitats. Since these ecosystems are especially sensitive to climate changes, as indicated by the worldwide retreat of glaciers and ice sheets as well as permafrost thawing, an understanding of the role and potential of microbial life in these habitats has become crucial. Emerging technologies have added significantly to our knowledge of abundance, functional activity, and lifestyles of microbial communities in cold environments. The current knowledge of microbial ecology in glacial habitats and permafrost, the most studied habitats of the cryosphere, is reported in this review.

## Introduction

The largest fraction of the Earth’s biosphere is exposed to temperatures below 5 °C throughout the year. Cold ecosystems are highly diverse and range from high mountains to deep oceans, polar region, and subterranean caves. They include aquatic and terrestrial ecosystems such as the deep sea (90% of the ocean volume is at a temperature below 5 °C), sea ice, lake ice, snow and glacial environments, cold water lakes, permafrost, cold soils and cold deserts, and even the atmosphere and clouds.

In recent years, studies on microorganisms in cold habitats has been mainly focused on the Earth’s cryosphere, i.e., frozen ecosystems, which include glaciers, ice sheets, sea ice, lake ice, and frozen ground (permafrost) (e.g., Anesio et al. 2017; Boetius et al. 2015; Hotaling et al. 2017a; Jansson and Tas 2014; Martin and McMinn 2018) and a recognition of the cryosphere as one of the biomes on Earth has even been proposed (Anesio and Laybourn-Parry 2012). Indeed, since ecosystems in the cryosphere are especially sensitive to climate changes (Beniston et al. 2018; Bibi et al. 2018; Huss et al. 2017), as indicated by the worldwide retreat of glaciers and ice sheets as well as permafrost thawing, an understanding of the role and potential of microbial life in these habitats has become crucial. Additionally, frozen ecosystems are also of much interest as analogues of extraterrestrial habitats (Cheptsov et al. 2017; Garcia-Lopez and Cid 2017; Jansson and Tas 2014) and as rich sources of various tools of promising biotechnological potential (Margesin 2017; Collins and Margesin 2019).

A better understanding of the role of microbial life in cold habitats has recently been attained by the incorporation of emerging new technologies (Aliyu et al. 2017; Jansson and Baker 2016; Nikrad et al. 2016; Raymond-Bouchard and Whyte 2017). In particular, metagenomics, the analysis of collective genomes of a microbial community in an environmental sample which gives information on gene presence and thus functional potential, has enhanced our knowledge significantly. As an example, about 1400 metagenome datasets were recently identified from cold environments (Aliyu et al. 2017). Nevertheless, a major limitation with metagenomic approaches lies in the uncertainty of the origin of the microbial community DNA, whether from living, dormant, or dead cells (Prosser 2015). Therefore, other –omic technologies have been developed to complement metagenomics. Among these, analysis of gene expression and proteomic analysis gives more insights into the mechanisms and metabolisms of microorganisms in cold habitats. Metatranscriptomics, the analysis of mRNAs, provides information on the regulation and expression profiles of complex microbiomes (Marchesi and Ravel 2015) and the analysis of numerous cold environments has added significantly to our knowledge of active functional groups in cold habitats. Over 20 transcriptomes are available for microorganisms from various cold habitats (Raymond-Bouchard and Whyte 2017). Similarly, metaproteomics, the large-scale characterization of the entire protein complement of an environmental sample at a given time point, provides an in-depth understanding of the molecular basis of biological processes in a given environmental sample and has allowed for the identification of many proteins involved in microbial cold adaptation (Kawamoto et al. 2017; Marchesi and Ravel 2015). Further insights into microbial function and growth in cold habitats has also been provided by stable isotope probing (SIP), which is based on DNA labeling of actively dividing microorganisms and allows for the estimation of taxon-specific population dynamics (Koch et al. 2018). Finally, nanoSIP, the combination of nanoscale secondary ion mass spectrometry (nanoSIMS)-generated information with molecular visualization methods, enables linking of in situ function with microbial identity and gene expression (Nunez et al. 2018).

In this review, the current state of cryomicrobiology in the two most studied cold habitats of the past 5 years, i.e., glacial habitats and permafrost, is summarized. Since it is not possible to cover all studies, the focus is on those reported mainly in the past five years. Regarding methodologies, most of the studies indicated here involve –omics approaches; however, other techniques such as SIP, FISH, and in situ studies are also included when they have contributed to significant advancements in the field.

## Psychrophilic lifestyle: molecular cold adaptation

Even the most extreme cold and frozen environments harbor enormously diverse and metabolically active microbial populations. Microorganisms in cold habitats (termed psychrophilic, cold-tolerant, or cold-adapted) face a number of challenges and often show multiple adaptations to the extremes (D'Amico et al. 2006). Besides low temperatures, in which physical and biological processes are slowed down, they may also have to cope with low nutrient conditions, high salinity, dryness, and low water activity, or even high UV irradiation and oxidative stress at high altitudes, and high pressure in the deep sea.

The true limiting factor for life in the cold is the lack of liquid water at low temperatures and not temperature per se (Bakermans 2017; Clarke 2014). Microbial reproduction occurs at subzero temperatures down to − 20 °C and metabolic activity has been detected down to − 30 °C. Interestingly, activities at significantly lower temperatures have also been documented but these await verification. The temperature limit for the completion of a life cycle in microorganisms depends on the cellular structure and is lower for unicellular than multicellular microorganisms ((Bakermans 2017; Clarke 2014; and refs. therein). The low-temperature limit for active metabolism (not just survival) of free-living unicellular microorganisms in the presence of external ice appears to be set by the vitrification (glass transition; a liquid behaves as a solid during cooling) temperature of the cytoplasm. This vitrification (analogous to the vitrification of a colloid) is mainly the result of freeze-induced desiccation and because of the high internal viscosity caused, movement of gases and metabolites, and thus metabolism, stops. However, in contrast to intracellular freezing, vitrification is not lethal and metabolism can start again once the cell warms and rehydrates (Bakermans 2017; Clarke 2014).

Microbial low-temperature adaptation requires a vast array of metabolic and structural adjustments at nearly all organization levels of the cell (Table 1). Considerable progress in our knowledge of molecular cold adaptation has been achieved by genomic, metatranscriptomic, and proteomic studies of psychrophiles (Barauna et al. 2017; Koh et al. 2017; Raymond-Bouchard et al. 2018a; Singh et al. 2014). Generally up-regulated functions for growth at low temperatures target the cell barrier (cell envelope biogenesis, membrane biogenesis, membrane-transport proteins, maintenance of membrane fluidity) metabolism (specific metabolic pathways, nutrient transport, energy metabolism), cell protection (production and uptake of cryoprotective compounds, antioxidant activities), protein synthesis, and folding (transcription, translation, RNA helicases, chaperones; cold shock, cold acclimation and heat shock proteins; adaptation of protein structures to ensure increased flexibility at low temperatures). Down-regulated genes are related to flagellar motility, outer membrane proteins and structures (flagella, chemotaxis), and pathways that produce reactive oxygen species. Comparative genomic, metatranscriptomic, and proteomic studies of permafrost bacteria growing at subzero temperatures and their mesophilic counterparts have revealed different features of cold adaptation (Raymond-Bouchard et al. 2018b) (Table 1), with both genome abundance (Dsouza et al. 2015) and reduced genome contents (Raymond-Bouchard et al. 2018b) being observed.

**Table 1:** Molecular adaptations of microorganisms in cold ecosystems (Barauna et al. 2017; D'Amico et al. 2006; De Maayer et al. 2014; Dsouza et al. 2015; Jansson and Tas 2014; Margesin and Miteva 2011; Margesin 2017; Raymond-Bouchard et al. 2018a; Siddiqui et al. 2013).

Target	Adaptation	Function
Morphology	Reduced cell size, intracellular membrane inclusions; modified appearance and composition of cell envelope (Mykytczuk et al. 2016; Perfumo et al. 2018)	Better efficiency of cell function
Cell movement	Chemotaxis, halotaxis, chemohalotaxis (Showalter and Deming 2018)	Colonization of sea ice
Cell membrane	Incorporation of unsaturated (branched and short) fatty acids in cell membranes; carotenoid pigment biosynthesis	Maintenance of membrane fluidity
Cell wall (outer cell membrane of Gram-negative bacteria)	Modification of lipopolysaccharides	Maintenance of membrane integrity and fluidity
Cell protection	Synthesis of cryoprotective molecules (antifreeze proteins, ice-nucleating proteins, compatible solutes, exopolysaccharides) (Cid et al. 2016; Perfumo et al. 2018)	Cell protection from freezing, desiccation, and hyper-osmolality
	Production of antioxidative enzymes (catalase, superoxide dismutase, dioxygen-consuming lipid desaturases)	Cell protection against reactive oxygen species to avoid membrane damage
	Production of carotenoids and mycosporins (Hassan et al. 2016)	UV protection
Proteins (enzymes)	Production of cold-active enzymes with increased structural flexibility	High activity at low temperature, low thermostability
Protein synthesis	Production of cold shock and constitutively expressed cold-acclimation proteins (Keto-Timonen et al. 2016)	Improved response to sudden temperature decrease; improved protein synthesis
Gene expression	Cold-inducible promotors (Singh et al. 2014)	Regulation of gene expression at low temperatures
Genome structure	Genome plasticity (rapid genome adaptation)	Increased flexibility
	Increased variety and number of chaperons; induction of peptidyl-prolyl-cis-trans isomerase to facilitate protein folding; modulation of RNA polymerase (Kawamoto et al. 2017)	Improved protein folding
	Increased variety and number of tRNA species; increased flexibility of tRNA (Lorenz et al. 2017)	Improved translation
	Mobile genetic elements (plasmids, transposons)	Genetic exchange
	*Exiguobacterium*: multiple stress-responsive genes: cold shock, heat shock, chaperones, carbon starvation, oxidative stress, detoxification, resistance to toxic compounds (Kasana and Pandey 2018)	Efficient stress response

## Microbial diversity and activity

### Glacial ecosystems

The characteristics of glacial ecosystems have been recently described in detail in a number of reviews (Anesio et al. 2017; Boetius et al. 2015; Hotaling et al. 2017b; Laybourn-Parry and Pearce 2016) and book chapters (e.g., Margesin 2017).

#### Supraglacial habitats

Supraglacial environments cover ~ 11% of the Earth’s surface area; they are seasonally or perennially covered by snow and are habitats for very diverse and metabolically active microbial communities (Edwards and Cameron 2017). The supraglacial zone is influenced by solar irradiation and amended with nutrients and microorganisms via atmospheric deposition (Hotaling et al. 2017b and refs. therein).

##### Snow

Snow covers permanently or seasonally the Earth’s surface covered by glacial ice and affects the climate due to its interaction with various biosphere compartments (atmosphere, soil, meltwater). A number of studies, with a focus on bacteria, have demonstrated a high diversity of metabolically active microorganisms in snow (reviewed by Edwards and Cameron 2017). Recently, comparative metagenomic analysis of Arctic (Choudhari et al. 2014; Maccario et al. 2014) and Antarctic (Lopatina et al. 2016) snow samples have been carried out. These showed that snow samples from an Alaskan glacier contained a rich and diverse microbial community of about 2500 bacterial species, with *Proteobacteria*, *Bacteroidetes*, and *Firmicutes* being the most abundant bacterial groups, and about 30 archaeal species. A large community overlap with Arctic soil was also found, indicating a strong involvement of the snow community in soil development during glacier retreat (Choudhari et al. 2014). Furthermore, the composition of the microbial communities in Arctic spring snow samples was shown to be impacted by photochemical reactions and oxidative stress (Maccario et al. 2014). On the other hand, communities in Antarctic snow samples were site-specific and contained a high variety of members of the genus *Flavobacterium*, which—probably as a result of a strong selection process—were only found in Antarctica and could not be detected in the Northern hemisphere (Lopatina et al. 2016).

Nitrate is often quantified in polar snow and ice as the major sink of atmospheric nitrogen oxides. Shi et al. (2018) studied nitrate deposition and preservation in 140 snow samples collected along a traverse from the coast to the ice sheet summit in East Antarctica and found that nitrate content increased from the coast to the summit and decreased with snow depth (i.e., were higher in surface snow samples). Furthermore, nitrate contents in inland surface snow were found to be influenced by gaseous deposition of nitric acid (Shi et al. 2018).

Snow algae (unicellular freshwater algae) have been described in polar and Alpine regions and appear to be cosmopolitan (Lutz et al. 2016; Segawa et al. 2018). The successful integration of metagenomics, targeted metabolomics, functional gene inventory, and identification of functional groups has contributed to the elucidation of the snow algae phenomenon in adjacent green and red snow algae fields on a glacier in Svalbard. It was demonstrated that the green and red snows are not successive stages, but occur independently and differ in their environmental characteristics, metabolic profiles, and algal community compositions. The wet and nutrient-rich green snow is colonized by *Microglena* sp. and contains metabolites involved in growth and proliferation, while storage metabolites and *Chloromonas* species are abundant in the dry and nutrient-poor red snow (Lutz et al. 2015).

According to a survey of microbial communities on 12 glaciers and snow fields, nitrogen limitation, rather than phosphorus limitation, plays a major role in the production of metabolites in snow and ice algae (Lutz et al. 2017). In contrast, isotopic and microcosm studies of supraglacial and periglacial snow algae communities on stratovolcanoes of the Pacific Northwest indicated dissolved inorganic carbon as the limiting nutrient and demonstrated active sequestering of Fe, Mn, and P leached from rocks (Hamilton and Havig 2017). This can be explained by the volcanic terrain delivering nutrients to snow and ice communities, in contrast to carbonate or granitic bedrock types.

##### Cryoconite holes

Cryoconite holes are “ice-cold hot-spots of microbial diversity and activity” (Edwards et al. 2013a) and biogeochemistry (Cook et al. 2016a). These water-filled ice depressions are found on glacier surfaces worldwide and are formed when dark particles like organic debris or inorganic dust deposited on the glacier surface are warmed by solar irradiation and sink into the ice. Due to the black body effect (accelerated thawing due to low albedo), cryoconite plays an important role in the acceleration of deglaciation processes. Cryoconite holes are microbe-mineral aggregates (Cook et al. 2016a, b) and rich in organic matter and are stable for several seasons in polar areas, in contrast to temperate mountain glaciers (Franzetti et al. 2017a). For a detailed literature survey on the physical, chemical, and biological properties of cryoconite and cryoconite holes, the reader is referred to Cook et al. (2016a).

The first metagenome from cryoconite was reported by Edwards et al. (2013b). This snapshot (i.e., without the consideration of temporal variability) of the phylogenetic and functional (see below) diversity of 14 Austrian Alpine cryoconite samples reported the dominance of bacteria (62% *Proteobacteria*) and a low abundance of eukaryotes (0.6%) and archaea (0.1%) (Edwards et al. 2013b). Network analysis of high-throughput DNA sequencing data detected that cryoconite holes host different and less diverse microbial communities than ice-marginal environments, such as moraines and glacier forefield, which differ in environmental conditions (temperature, solar irradiation, water availability) from cryoconite. For example, cyanobacteria were the most abundant taxon in cryoconite holes, but the least abundant taxon in ice-marginal environments. This shows that microbial communities not adapted to the specific environment may be sorted out and the composition of cryoconite communities may be driven by species-sorting processes (Franzetti et al. 2017a).

Both heterotrophic and autotrophic microbial communities are active in cryoconite. The ecological importance of phototrophs, principally cyanobacteria, as primary colonizers and net producers in supraglacial habitats is well known. Biogeographical analyses of cyanobacteria in cryoconite pointed to an endemic distribution of most of the 16S-23S ITS (internal transcribed spacer) derived phylotypes, attributed to geographically different selection pressures (Segawa et al. 2017). Using non-targeted metabolomics, it was shown that microbial processes contribute to the formation and maintenance of stable autotrophic conditions in cryoconite holes. Furthermore, cryoconite holes can change their shape in response to changes in light intensity and sediment thickness (Cook et al. 2016b). Irregular shape of cryoconite holes was interpreted as migration away from shady topographic settings over ice surfaces towards more illuminated and flat areas on ice (Cook et al. 2018). Indeed, the authors hypothesized the movement from net heterotrophy to net autotrophy with the promotion of carbon fixation; this however has not been confirmed.

Biodiversity studies conducted over three summer months in 60 Alpine cryoconite samples demonstrated temporal shifts between autotrophic cyanobacteria, dominating after snowmelt, and heterotrophic *Sphingobacteriales*, being more abundant later in the season. Interestingly, sampling time (July, August, and September 2013) had a higher influence on the composition of bacterial communities than the local environmental conditions prevailing in each cryoconite sample (Franzetti et al. 2017b). Metagenomic analysis of cryoconite on Himalayan and Alpine glaciers revealed a high abundance of heterotrophic anoxygenic phototrophic *Proteobacteria* (Franzetti et al. 2016). These bacteria use light on the glacier surface as an energy source to produce biomass from organic carbon of allochthonous origin, e.g., chitin. Alternatively, organic carbon would be converted by other microorganisms via heterotrophic respiration to CO_2_, which then would be converted to organic carbon by cyanobacteria via oxygenic photosynthesis (Franzetti et al. 2016).

RNA-based studies of microbial communities are used to evaluate potentially active organisms, which is not possible with DNA-based studies. However, rRNA is not a general indicator of microbial activity in environmental samples and should rather be seen as an indicator of protein synthesis potential (Blazewicz et al. 2013). Furthermore, rRNA:rRNA gene ratios are not reliable indicators for differentiating between active and inactive microbial taxa in environmental samples. In fact, the relationship between rRNA concentration and growth rate is very variable and differs, often significantly, among taxa. In addition, the presence of high numbers of ribosomes in dormant cells may result in an overestimation of microbial activity in environments with a high abundance of dormant microorganisms (Blazewicz et al. 2013). The comparison of bulk (rDNA) and potentially active (rRNA) bacterial communities in Greenland cryoconite revealed a higher abundance of potentially active bacteria (*Cyanobacteria*, *Proteobacteria*) in the interior than at the margin of the ice sheet (30-km distance), while diversity was higher in the margins (Stibal et al. 2015). Elevated nitrate levels, simulated in line with the predicted tropospheric nitrate content for the year 2100 (a 181% increase), had no significant effect on abundance and little effect on the composition of bulk or potentially active microbial communities in a 6-week in situ study using N-isotopes (Cameron et al. 2017).

Heterotrophic microorganisms in Himalayan and Antarctic cryoconite holes had a high potential to metabolize a range of organic compounds (carbohydrates, lipids, proteins, cellulose, lignin) and to produce extracellular enzymes (Sanyal et al. 2018). Biomarker and stable isotope analyses of organic carbon indicated that microorganisms in cryoconite do not use geological (aged) organic carbon, and that their primary source of carbon (carbon fixation) is recent (fresh) atmospheric carbon (McCrimmon et al. 2018). In microcosm-experiments, 13–60% of dissolved organic carbon in Himalayan and Antarctic cryoconite holes was bioavailable to heterotrophic microorganisms (Sanyal et al. 2018). Thus, microorganisms in cryoconite play an important role in carbon turnover (carbon cycling and fixation). During melting and flushing of cryoconite holes (glacial runoff), microorganisms and high amounts of microbially produced dissolved organic carbon are exported downstream, contributing to carbon cycling processes in glacial forefields, subglacial ecosystems, and coastal waters (Edwards et al. 2014; Sanyal et al. 2018).

The analysis of (metagenome-derived) functional genes, indicating the functional potential, in 14 Alpine cryoconite samples and a comparison with 32 metagenomes from comparable environments showed a high abundance of genes linked to the utilization of carbohydrates and amino acids, while genes linked to photosynthesis and dormancy/sporulation were low. The presence of genes associated with various nutrient cycles (N, S, Fe, P), with a trend towards assimilative metabolism, indicated an efficient microbial nutrient uptake. A preference for ammonia utilization compared to denitrification and N fixation, and the importance of phosphate, organic, and inorganic sulfur, Fe(II) and Fe(III) as nutrient sources were evident (Edwards et al. 2013b). Fe enrichment in cryoconite water was recently shown to exceed enrichment in marine phytoplankton, suggesting that supraglacial habitats are a source of Fe to downstream processes (Fortner and Lyons 2018).

Viral-induced lysis and lowering of the growth efficiency of bacteria is a dominant process for the release and recycling of carbon and nutrients in Arctic cryoconite holes (Bellas et al. 2013). Thus, viruses alter carbon cycling substantially, which impacts nutrient cycling (Rassner 2017). Some bacteria have developed defense mechanisms against viral attack. *Janthinobacterium* (belonging to *Betaproteobacteria*), a dominant bacterium in supraglacial meltwater and often found in cold habitats, produces vesicles on the outer membrane. These vesicles contain the binding sites recognized by the specific virus. In case of a viral attack, the virus cannot distinguish between host cells and host vesicles, binds to the vesicles and injects the genome into the vesicle, which prevents its replication (Rassner 2017; Rassner et al. 2016). In this way, this dominant bacterial taxa evades viral control.

Viruses in cryoconite and cryoconite holes have been reported in a number of studies (Rassner 2017 and ref.s therein). A study of viral content in Arctic cryoconite samples in Greenland and Svalbard showed a tenfold higher abundance of viruses compared to bacteria and a virus production (up to 9 × 10^7^ g dry mass) comparable to that in sediments worldwide (Bellas et al. 2013). Novel dsDNA virus genomes with genes to control host genomes were found in the cryoconites. The majority of viruses (bacteriophages, cyanophages) belonged to *Caudovirales* (bacteriophages) and the most prominent bacterial hosts were members of *Alpha*- and *Gammaproteobacteria*, *Actinobacteria*, *Firmicutes*, and *Cyanobacteria*. Some viral groups were found to have unusual life strategies and possess genes to control host replication (Bellas et al. 2015). Importantly also, glacial viruses are characterized by longevity and can adapt to the cold separately from the host (Rassner 2017).

#### Subglacial habitats

The characteristics of subglacial environments, including subglacial lakes, have been described in detail in a number of reviews (e.g., Hotaling et al. 2017b) and book chapters (Achberger et al. 2017). Since access to these often remote areas is difficult for technological, financial, and environmental reasons, knowledge of microbial life in subglacial habitats is still limited. The development of microbiologically clean core-drilling techniques, such as hot water drilling (Hodgson et al. 2016; Makinson et al. 2016; Priscu et al. 2013), and the application of radioglaciology (the study of glaciers and ice sheets using radar) have resulted in the detection and exploration of several polar subglacial lakes and allowed the detailed study of microbial diversity and metabolic activity (Achberger et al. 2017; Bulat 2016). Interestingly, despite several limiting conditions (permanently cold temperatures, darkness, low amounts of available nutrients), metabolically active chemolithoautotrophic and heterotrophic microorganisms have been found in all subglacial habitats examined so far (basal ice, sediment cores, subglacial lakes, subglacial outflows at glacial margins) (Achbergert et al. 2016; Mikucki et al. 2016; Vick-Majors et al. 2016).

Biodiversity and functional gene studies in subglacial habitats have been recently reviewed by Achberger et al. (2017) and are described here only briefly. Analyses of metagenomes (DNA, RNA, isolates) and of 16S rRNA genes and transcripts indicated the dominance of bacteria (*Proteobacteria*, *Firmicutes*, *Actinobacteria*, *Bacteroidetes*), while archaeal and eukaryotic members were rarely found in the subglacial Antarctic Lake Vostok (Bulat 2016) and in water and sediments of the subglacial Antarctic Lake Whillans (Achbergert et al. 2016). Active methane cycling, as demonstrated by the presence of methanotrophic and methanogenic taxa and methane monooxygenase (*pmoA*) transcripts, as well as the measurement of methane oxidation, was reported in subglacial water of the Greenland Ice Sheet (Dieser et al. 2014). The potential for methane oxidation was also detected beneath the Antarctic Ice Sheet (Michaud et al. 2017). The oxidation of reduced iron, sulfur, and nitrogen by chemolithoautotrophic microorganisms has also been shown in this area (Achbergert et al. 2016; Purcell et al. 2014) as well as in glacier sediments in the Canadian Rockies (Boyd et al. 2014). The metabolic activity of these microorganisms was confirmed via SIP (e.g., Boyd et al. 2014; Michaud et al. 2017) and microcosom/culture-based studies (e.g., Harrold et al. 2016) and the presence of active sulfide-oxidizing microorganisms was confirmed by 16S rRNA transcripts closely related to *Thiobacillus* and *Sideroxydans* spp. (Achbergert et al. 2016; Michaud et al. 2016). Such studies indicate that microbial chemolithotrophy plays an important role in biogeochemical processes in subglacial ecosystems (Anesio et al. 2017).

### Frozen ground

Research on permafrost has considerably increased in recent years due to emerging concerns about the impacts of climate change (global warming) and subsequent permafrost thawing on the activation of indigenous microorganisms. The availability of trapped organic carbon and other nutrients (nitrate) after thawing is likely to increase microbial activity, which results in an increased release of greenhouse gases from the biosphere to the atmosphere (Hultman et al. 2015; Mackelprang et al. 2016). In Alpine regions, permafrost thawing can affect the geotechnical stability of mountain permafrost as shown by recent rockfalls in the European Alps. Microbial responses to climate warming are still not fully understood. However, the development and application of a number of sophisticated techniques to permafrost ecosystems, such as next generation sequencing combined with various –omics approaches, SIP, and in situ studies, has allowed for demonstration of the presence and subzero temperature activity of a wide diversity of microorganisms while also enabling the study of their adaptive mechanisms to frozen conditions (Nikrad et al. 2016; Raymond-Bouchard and Whyte 2017).

#### Terrestrial permafrost

Vast areas (ca. 25%) of the terrestrial surface on Earth are underlain by permafrost. Permafrost is defined as ground (lithosphere: soil, sediment, rock), which is permanently exposed to temperatures ≤ 0 °C and has remained frozen for at least two consecutive years. Permafrost-affected soils are seasonally (summer) thawed soils (active layers) underlain by permafrost. Permafrost soils contain 20–70% ice and 1–7% unfrozen water with a low water activity (0.8–0.85). Further characteristics that impact microbial life are oligotrophic conditions, constant darkness, and geological background gamma-radiation (Margesin 2009).

Permafrost regions occur at high latitudes and at high elevations. Research on permafrost microbiology has focused for many years on polar regions, while mountain permafrost has been largely neglected. Permafrost at high elevations occurs in the Alps, Andes, and Himalayas and is characterized by higher spatial variability than polar permafrost, higher mean temperatures, and a lower influence of vegetation (Frey et al. 2016; Hu et al. 2015). Arctic permafrost is characterized by a mean temperature of − 10 °C and low content of organic carbon and nitrogen, while Antarctic permafrost is subjected to much lower temperatures (− 18.5 to − 27 °C) and alkaline pH. Polar and mountain permafrost characteristics have been described in detail in various reviews (e.g., Jansson and Baker 2016; Jansson and Tas 2014) and book chapters (Altshuler et al. 2017; Margesin 2009).

Permafrost bacteria are often very small in size (dwarfed, ≤ 1 μm) and viable but non culturable (Margesin 2009). Culture-independent methods and the combination of various –omics approaches revealed a higher and different bacterial diversity compared to culture-dependent techniques (Hultman et al. 2015). Generally, the by far most abundant group is the bacteria. Archaea (often methanogenic and halophilic *Euryarchaeota* and *Crenarchaeota*) and fungi (with a prevalence of *Ascomycota*) have also been detected, at much lower abundances, both in polar and Alpine permafrost (Altshuler et al. 2017; Hu et al. 2015; Jansson and Tas 2014). Cyanobacteria were not detected in Arctic permafrost samples, while they were present but unculturable in Antarctic permafrost (Makhalanyane et al. 2015).

It has been often assumed that microorganisms in permafrost are in a dormant state; however, a number of studies have evidenced replication and active metabolism under frozen conditions (Nikrad et al. 2016; Vishnivetskaya et al. 2018). For example, bacterial genome replication in permafrost soils, determined by SIP via the incorporation of ^13^C-acetate into DNA in a 6-month microcosm study, was detected over a temperature range of 0 to − 20 °C for a number of bacterial phyla (*Actinobacteria*, *Acidobacteria*, *Proteobacteria*, *Chloroflexi*, *Gemmatimonadetes*) with the exception of *Firmicutes*. Interestingly, some bacteria were only able to replicate at temperatures lower than − 6 °C (Tuorto et al. 2014) and 16S rRNA sequencing demonstrated a greater diversity of active OTUs at subzero temperatures than at 0 °C (Tuorto et al. 2014). According to a proteomic study, subzero growth (− 10 °C) of *Planococcus halocyrophilus* from permafrost resulted in a series of complex responses and significant changes in the abundance of proteins involved in various cellular processes, such as synthesis of cell wall, fatty acids, carotenoids, amino acids, energy metabolism, nucleotide turnover, transcription, translation, and DNA replication and repair (Raymond-Bouchard et al. 2017). This bacterium is able to grow over an exceptionally wide temperature range, from − 15 to 37 °C, and modifies peptidoglycan metabolism (by reducing active peptidoglycan synthesis) and the iron acquisition strategy (by omitting siderophore-mediated iron and preferring ABC-type iron acquisition) after transition from optimal (24 °C) to subzero temperatures (Ronholm et al. 2015). Also, when grown at − 15 °C, its cell envelope differed from that of cells grown at 25 °C by the accumulation of high contents of calcium carbonate (due to biomineralization) and choline, which could represent cryoprotective mechanisms at subzero temperatures (Mykytczuk et al. 2016). Indeed, these surfaceomics studies are an interesting approach to evaluate the impact of subzero temperatures on microbial adaptation strategies. Similarly, comparative genomics has also enabled a better understanding of microbial adaptation, with, for example, comparison of the genome of *Rhodococcus* sp. JG3, isolated from Antarctic McMurdo Valley, with 14 mesophilic counterparts revealing a number of survival strategies in permafrost; including lipid alteration, osmotic stress response, nutrient acquisition via the utilization of reserve molecules, and carbon utilization via the full glyoxylate pathway (Goordial et al. 2016b).

Generally, metabolic activities involved in the cycling of carbon (degradation of carbon compounds, methanogenesis, methanotrophy), nitrogen (nitrification, denitrification, nitrogen fixation), sulfur (sulfate and thiosulfate reduction), and iron (Fe(III) reduction) have been evidenced in permafrost in a number of studies by the presence of the respective genes, proteins, and transcripts (Altshuler et al. 2017). Studies have also indicated that archaeal methanogens may be enriched in microcosms of Antarctic Dry Valleys permafrost sediments and the metagenome-assembled genome of an unculturable *Methanosarcina* representative was shown to contain cold-active enzymes and metabolic pathways (Vishnivetskaya et al. 2018). An excellent summary of metatranscriptome studies in permafrost and permafrost-affected environments is given by Raymond-Bouchard and Whyte (2017). A multi-omics study (Hultman et al. 2015) of microbial processes and phylogenetic composition in Alaskan permafrost was based on metagenomics, metatranscriptomics, and metaproteomics. In this study, the analysis of nucleic acid ratios (RNA:DNA; functional gene transcripts to genes) identified *Proteobacteria*, *Acidobacteria*, and *Firmicutes* as the most active members of microbial communities, acclimated for activity at subzero temperatures. Furthermore, different microbial strategies in permafrost and seasonally thawed active layers were demonstrated. Permafrost microorganisms were characterized by low process rates, applied dissimilatory Fe(III) reduction as a potential metabolic strategy, and had a lower general functional potential than thawed soils. However, the relative abundance of transcripts and proteins for some pathways, such as methane oxidation, was comparable in permafrost and active layers. In addition, microbial communities in the active layer expressed genes and proteins necessary for energy and nutrient supply and for survival under freeze-thaw conditions (Hultman et al. 2015).

A very diverse and largely under-characterized permafrost microbiome was described in a comprehensive diversity analysis of all three microbial domains in high alpine permafrost located at 2979 m above sea level in Switzerland (Frey et al. 2016). In contrast to studies at high latitudes, where deeper permafrost layers are often compared with the overlaying active layer, soils at the same depths on two slopes were compared, i.e., permafrost soils at the NW slope and non-permafrost soils at the SE slope. High-throughput sequencing of ribosomal markers detected the enrichment of unculturable bacteria in the permafrost, especially members of the candidate superphylum *Patescibacteria* (phyla OD1 *Parcubacteria*, TM7 *Saccharibacteria*, GN02 *Gracilibacteria*, OP11 *Microgenomates*). Interestingly, these bacteria were found to be characterized by small streamlined genomes and anaerobic fermentative metabolism and this combination appears to be the key for microbial survival and adaptation in alpine permafrost (Frey et al. 2016 and refs. therein).

Environmental conditions determine the strategies of microbial communities to cope with the cold. Permafrost soils at lower elevations in the McMurdo Dry Valleys of Antarctica contained active microbial populations, while those at higher elevations had a high richness of genes involved in dormancy and sporulation and a low richness of stress response genes and thus favor survival or dormancy (Goordial et al. 2016a, 2017). In contrast, cryptoendolithic communities (colonizing cavities in porous rock) in porous sandstone at higher elevations were metabolically active at in situ temperatures and had a high richness of genes involved in metabolic activity (carbon fixation) and stress response, which are needed for growth under the prevailing oligotrophic, arid, and cold conditions (Goordial et al. 2017). Organic biomarker and radiocarbon analyses indicated that these crypotendolithic microbial communities are active and recycle carbon on timescales faster than previously assumed (Brady et al. 2018).

#### Thawing permafrost

Permafrost thawing results in increased microbial activity and consequently increased emission of greenhouse gases such as CO_2_, CH_4_, and N_2_O. The effect of permafrost thawing on microbial communities and their activities has thus become an important research topic (Graham et al. 2012; Mondav et al. 2017).

Increased methane emission after permafrost thawing resulted in a tenfold increase in the abundance of methanogenic archaea and in significant shifts in archaeal community composition and their activities. The dominance of *Methanosarcinales* with increased transcriptional activities in thawed permafrost indicates a shift of metabolic processes of methanogens from hydrogenotrophic to partly acetoclastic methane production (Wei et al. 2018). A metatranscriptomic study on metabolic pathways in up to 70 cm deep Alaskan permafrost soils before and 11 days after thawing showed a rapid activity increase (enzymes, carbohydrate metabolism, methanogenesis) after thawing, resulting from the decomposition of soil organic matter by *Bacteroidetes*, *Firmicutes*, *Ascomycetes*, and methanogens. The exclusive expression of transcripts involved in acetogenesis after thawing indicated acetogenic bacteria as the potential acetate source for acetoclastic methanogens in freshly thawed permafrost (Coolen and Orsi 2015).

Thawing permafrost results in the formation of lakes and ponds which are a source of emission of CO_2_ and CH_4_ to the atmosphere. A study of aerobic methanotrophic bacteria in such ponds pointed to the resilience of methanotrophs (mainly *Methylobacter*) and their methanotrophic potential activity (pmoA transcripts), regardless of oxygen content (Crevecoeur et al. 2017). Field and laboratory methane flux measurements, combined with –omics approaches, demonstrated the presence of active atmospheric methane-oxidizing (methanotrophic) bacteria in permafrost-affected Arctic mineral (carbon-poor) cryosols (Lau et al. 2015). Atmospheric methane oxidation in microcosms of these soils increased with increasing temperature (10 °C versus 4 °C), but decreased with decreasing water saturation and soil depth, independent of temperature. According to stable isotope mass balance, ca. 50% of the oxidized methane was incorporated in microbial biomass, regardless of temperature, water saturation, or soil depth (Stackhouse et al. 2017).

SIP was applied to study the processes responsible for high N_2_O emissions from subarctic permafrost peatlands (Gil et al. 2017) and identified denitrification as the main process responsible for increased N_2_O emission to the atmosphere as a consequence of global warming. SIP was also used to demonstrate enhanced microbial nitrification, due to the release of nitrogen (nitrate), after permafrost disturbance in the Canadian High Arctic (Louiseize et al. 2014).

Soil structure plays an important role in long-term storage of organic carbon. A combination of physical fractionation, reflected light microscopy, scanning electron microscopy, and NanoSIMS was used to investigate soil structure at the microscale in a cross section from an Arctic permafrost layer in Alaska (Mueller et al. 2017). It was shown that plant residues, accounting for 58% of the organic carbon stored in the studied soil section (80–126-cm depth), act as nuclei for the formation of microaggregates composed of mineral-associated organic matter. Such microaggregates are important for organic matter preservation in soils, which is of relevance for the deepening of active layers of permafrost-affected soils in the context of warming.

#### Submarine permafrost

In contrast to land permafrost, submarine permafrost is not only subjected to heat transfer, but also to the penetration of salt from the ocean water, which lowers the freezing temperature (Mitzscherling et al. 2017). The effect of thawing of submarine permafrost on microbial communities has been poorly investigated. A recent study identified anaerobic archaeal methanotrophs of marine and terrestrial origin in frozen and thawed deep submarine permafrost (Winkel et al. 2018). Here, potential activity of anaerobic methane-oxidizing communities at low temperatures was revealed by using functional marker genes (*mcrA*) and catalyzed reporter deposition fluorescence in situ hybridization (CARD-FISH). The modeling of potential methane oxidation activity predicted the consumption of 72–100% of submarine permafrost methane, which the authors proposed for consideration in global methane budgets (Winkel et al. 2018). In another study, the investigation of two submarine permafrost cores from the East Siberian Arctic shelf, flooded 540 and 2500 years ago, resulted in the detection of stratified site-specific bacterial communities in permafrost, marine-affected permafrost and seabed permafrost. Interestingly, the ice-bonded seawater of the 2500 years inundated core, unaffected by warmed permafrost, contained the highest microbial abundance. The authors concluded that proliferation of submarine permafrost bacterial communities starts only millennia after warming (Mitzscherling et al. 2017).

## Conclusions and future perspectives

There is an increasing interest in studies of the microbial ecology of cold ecosystems, especially frozen ecosystems such as glacial habitats and frozen ground. Such an interest in understanding the role and behavior of microorganisms in these habitats is mainly related to the sensitivity of these environments to climate changes. The rapid development of emerging new technologies for the analysis and characterization of microorganisms, the incorporation of these technologies in studies of microbial life in cold habitats, and their complementation with well-established “traditional” methods have enhanced our knowledge of these environments significantly, especially with regard to gene presence, functional gene potential, gene expression, and in situ identification of active microorganisms. Reduced sequencing costs and the availability of further genomes from different taxa will soon also give better insights into the evolution of cold adaptive characteristics and allow for the identification of taxa-specific cold adaptations (Goordial et al. 2016b). Indeed, this should also result in an enhanced application of cold-adapted microorganisms and their cellular components in biotechnology (Collins and Margesin 2019). More fundamental ecological research is still however urgently needed. The more we know, the better, earlier, and more appropriately we can react to microbial responses to climate changes in cold ecosystems. However, this is associated with a number of challenges and outstanding questions, as indicated below, and future studies should be focused on addressing these.

- At present, there is a limited ability to predict, and manage, the dynamics and functions of microbial communities. Empirical approaches (field and laboratory studies) currently dominate the study of microorganisms in cold habitats while ecological modeling approaches, such as recently reported for the supraglacial ecosystem (Stibal et al. 2017), are rare. Progress towards a better fundamental understanding of microbial communities in the cold could be achieved in the near future by the development of predictive mathematical models to complement the empirical studies (Bradley et al. 2016; Widder et al. 2016). Such an approach would require increased interdisciplinarity, linking theory and experiment.
- Studies on interactions between micro- and macroorganisms in cold habitats, such as feedback between tropic levels, are currently underrepresented. In addition, there is a need for more extensive studies on interactions between (micro)biology and environmental conditions (temperature), which should include microhabitats and microclimates.
- Climate change represents a threat to biodiversity, especially in cold habitats. Linkage of biodiversity, climate, and function is an important emerging question.
- The role and behavior of cold-adapted microorganisms in various processes associated with climate warming (e.g., deglaciation, permafrost thawing) is far from being clear. To answer this question (over the long-term time range), an integrative view of all involved aspects (spatial and temporal, chemical, and physical effects, interactions between micro- and macroorganisms) is required.
